# Visualization of tumor-related blood vessels in human breast by photoacoustic imaging system with a hemispherical detector array

**DOI:** 10.1038/srep41970

**Published:** 2017-02-07

**Authors:** M. Toi, Y. Asao, Y. Matsumoto, H. Sekiguchi, A. Yoshikawa, M. Takada, M. Kataoka, T. Endo, N. Kawaguchi-Sakita, M. Kawashima, E. Fakhrejahani, S. Kanao, I. Yamaga, Y. Nakayama, M. Tokiwa, M. Torii, T. Yagi, T. Sakurai, K. Togashi, T. Shiina

**Affiliations:** 1 Department of Breast Surgery, Graduate School of Medicine, Kyoto University, 54 Shogoin-Kawaharacho Sakyo-ku, Kyoto 606-8507, Japan; 2 Medical Imaging System Development Center, Canon Inc., 3-30-2 Shimomaruko, Ohta-ku, Tokyo 146-8501, Japan; 3 Department of Diagnostic Imaging and Nuclear Medicine, Graduate School of Medicine, Kyoto University, 54 Shogoin-Kawaharacho Sakyo-ku, Kyoto 6068507, Japan; 4 Department of Diagnostic Pathology, Graduate School of Medicine, Kyoto University, 54 Shogoin-Kawaharacho Sakyo-ku, Kyoto 606-8507, Japan; 5 Department of Human Health Science, Graduate School of Medicine, Kyoto University, 53 Shogoin-Kawaharacho Sakyo-ku, Kyoto 606-8507, Japan

## Abstract

Noninvasive measurement of the distribution and oxygenation state of hemoglobin (Hb) inside the tissue is strongly required to analyze the tumor-associated vasculatures. We developed a photoacoustic imaging (PAI) system with a hemispherical-shaped detector array (HDA). Here, we show that PAI system with HDA revealed finer vasculature, more detailed blood-vessel branching structures, and more detailed morphological vessel characteristics compared with MRI by the use of breast shape deformation of MRI to PAI and their fused image. Morphologically abnormal peritumoral blood vessel features, including centripetal photoacoustic signals and disruption or narrowing of vessel signals, were observed and intratumoral signals were detected by PAI in breast cancer tissues as a result of the clinical study of 22 malignant cases. Interestingly, it was also possible to analyze anticancer treatment-driven changes in vascular morphological features and function, such as improvement of intratumoral blood perfusion and relevant changes in intravascular hemoglobin saturation of oxygen. This clinical study indicated that PAI appears to be a promising tool for noninvasive analysis of human blood vessels and may contribute to improve cancer diagnosis.

Angiogenesis is essential for organ growth and repair, and is strongly affected by the microenvironment of cancer tissues^1^,^2^. Therefore, a variety of imaging modalities have been used in order to analyze the tumor-associated vasculatures. However, most of them need contrast medium injection for imaging and the spatial resolutions wouldn’t be sufficient enough. Optical detection of breast cancer using near-infrared light has drawn considerable attention because it can noninvasively measure the distribution and oxygenation state of hemoglobin (Hb) inside the tissue^3^,^4^. Several clinical studies have reported promising results from using diffuse optical tomography (DOT) for the differential diagnosis and therapeutic monitoring of breast cancer^5^,^6^,^7^. DOT overcomes some of the drawbacks of conventional diagnostic modalities, such as X-ray mammography (XMMG), ultrasonography (US), and magnetic resonance imaging (MRI): XMMG is associated with ionizing radiation and has lower diagnostic sensitivity for dense breast tissue; the results of US depend greatly on instrumental performance and the examiner’s skill; and MRI requires the use of contrast medium, which is not suitable for mass screening or repeated examination. However, the clinical usefulness of optical detection has been limited by poor spatial resolution.

Photoacoustic tomography (PAT), which is based on photoacoustic (PA) technology, is another type of optical imaging that can image the distribution and oxygenation state of Hb with higher spatial resolution than DOT. The use of this approach for human breast diagnosis—a technique known as photoacoustic mammography (PAMMG)—has been explored in many theoretical analyses and phantom measurements, and a few preliminary reports. Four groups have reported detection of breast cancer lesions using PAMMG compared with XMMG and/or MRI: Manohar *et al*. and Heijblom *et al*. described a PAMMG system with a flat-shaped static detector array and a light delivery system providing illumination from the cranial direction^8^,^9^,^10^; Kitai *et al*. and Fakhrejahani *et al*. reported clinical results obtained with a dual illumination-mode PAMMG, which was called PAM-01 prototyped by our group, that used a flat-shaped scanning detector and a light delivery system providing illumination from both the cranial and caudal directions^11^,^12^. Although it is important to compare images obtained by PAMMG with those obtained by standard methods, breast shape and position must be carefully considered because of the different means of breast positioning used in each approach. Asao *et al*. reported PAMMG capable of simultaneously acquiring photoacoustic and ultrasound images, which was called PAM-02 prototyped by our group and was inherited from the PAM-01 configuration for the PA image and equipped with simultaneously acquiring US image^13^. The detector arrays introduced by the above two groups were flat shaped and it is considered that they had drawbacks of a limited view^14^. Ermilov *et al*. described a PAMMG system with a cylindrical-shaped detector array^15^. Li *et al*. described a PAMMG system with a ring-shaped detector array^16^. It is thought that they have a similar limited view issue since the detector arrays were flatly arranged in the direction orthogonal to the bottom surface of the cylinder or the direction perpendicular to the ring surface.

Kruger *et al*. reported breast imaging of four healthy women volunteers using a PAMMG system with a hemispherical-shaped detector array (HDA)^17^. This system was still affected by a limited view to some extent; however, they reported that it allowed the collection of nearly spatially isotropic three-dimensional reconstructed images of blood vessels. Although use of this system is feasible as the vascular networks seem to be clearly imaged, there is little disclosure on the image depth in the actual breast tissue because of reporting only in the MIP image and also it does not provide morphological information regarding the tumor mass.

In this study, we adopted the PAMMG system with HDA (hereinafter referred to as PAM-03) and applied the breast deformation algorithm from the breast shape in an MR image to that in a PA image to analyze the morphology and function of tumor-related blood vessels. We report the clinical findings of imaging tumor tissue and tumor-related blood vessels in patients with breast cancer.

## Results

### Pre-clinical study

We performed phantom measurements as described in a previous report^17^, and confirmed the reproducibility of the contrast-to-noise ratio and spatial resolution.

Additionally, we performed SO_2_ measurements on a simulated manner using a wire-shaped phantom (0.3 mm in diameter, 80 mm in length) that emulated the absorbance spectrum of Hb at the wavelengths used in PAM-03. We confirmed that the magnitude relationship of measured SO_2_ values was maintained between each phantom and across the absorption spectrum. The SO_2_ value as measured by PAMMG is a relative index called the “S-factor”^13^.

### Performance assessment on non-diseased breasts

**Figure 1:**
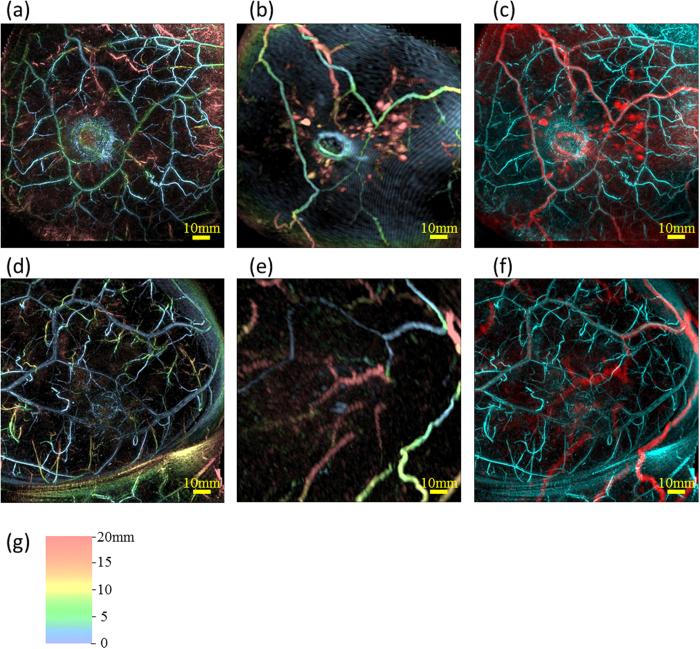
Examples of breast deformation of MRI and comparison of visibilities of blood vessels between PAI and MRI using maximum intensity projection (MIP) images on the healthy breast. Case 1 (Fig. 1(a–c)): (**a**) PA image, (**b**) MR image deformed to correspond to the PA image, and (**c**) fusion image of PA (cyan) and MR (red). Case 2 (Fig. 1(d–f)): (**d**) PA image, (**e**) MR image deformed to correspond to the PA image, and (**f**) fusion image of PA (cyan) and MR (red). All images are coronal views. In Fig. 1(a), (b), (d) and (e), we colored the signals according to the depth using the color chart shown in Fig. 1(g). This color chart for depth information is also used in Figs 2, 4 and 5.

We assessed the performance of PAM-03 on the healthy breasts contralateral to the affected breast of patients in this clinical study. First, we tested the feasibility of applying the breast shape deformation of MRI to PAI. Deformation of the MR image was carried out by manually selecting corresponding points between the PA and MR images, as defined by landmarks such as blood-vessel branching points. Figure 1(a–f) show examples of deformation of an MR image to correspond to a PA image on two cases (Case 1 and 2). The shapes and positions of blood vessels after the breast shape deformation of MRI were in good agreement with PAI by visual examinations as shown in Fig. 1(c and f). The precision of image deformation was estimated by the leave-one-out method. In these two cases, the corresponding point count and accuracy of the breast deformation were 161 and 1.9 mm in Case 1, and 65 and 2.6 mm in Case 2, respectively. In addition, PAI revealed finer vasculature, a more detailed blood-vessel branching structure, and more detailed morphological vessel characteristics compared with MRI as can be seen in Fig. 1(c and f).

**Figure 2:**
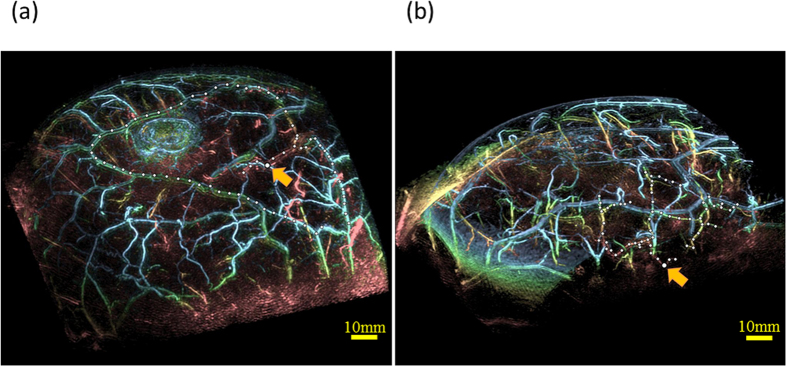
Examples of MIP images of the bird view illustrating depth performance. The color of PA signal corresponded to the depth scaled by Fig. 1(g). The linear PA signal marked by white filled circles was considered to be the identical blood vessel judging from the continuity of the PA signal. Slightly bigger white filled circle indicated by the orange arrow was the deepest point from the skin surface in these circles. Estimated depths from skin were (**a**) 24 mm in Case 1 and (**b**) 27 mm in Case 2. (See Supplemental Multimedia Files).

Next, we assessed the depth performance. Examples of maximum intensity projection (MIP) images obtained by PAM-03 are given in Fig. 2. Although MRI signals of narrow blood vessels were few or none in the breast, we assessed the depth performance from the significant PA signal which was considered to be the blood vessel by the continuity from the subcutaneous blood vessel. Filled circles were plotted on the continuous linear signal and yellow arrow was the deepest signal in each example. The depth of yellow arrow was estimated to be a 24 mm in Fig. 2(a) and 27 mm in Fig. 2(b), which were the evaluation results of the depth in the state where compression by the breast cup existed to some extent.

### Clinical study of patients with breast tumors

We performed PAM-03 measurements on patients with breast tumors, including benign (fibroadenoma) and malignant disease. Of the 30 patients considered for the study, five were not scanned for personal reasons or because of technical problems with the PAMMG machine. One case that had asthma and thus could not receive MRI contrast agent was omitted, as was one case in which no lesion was identified by MRI. The 22 malignant cases were scored, and results were compared between the five cases of ductal carcinoma *in situ* (DCIS) and the 17 cases of invasive breast carcinoma (IBC), which included one case of invasive lobular carcinoma (ILC) and 16 cases of invasive ductal carcinoma (IDC). The one benign case was excluded from quantitative analysis.

**Figure 3:**
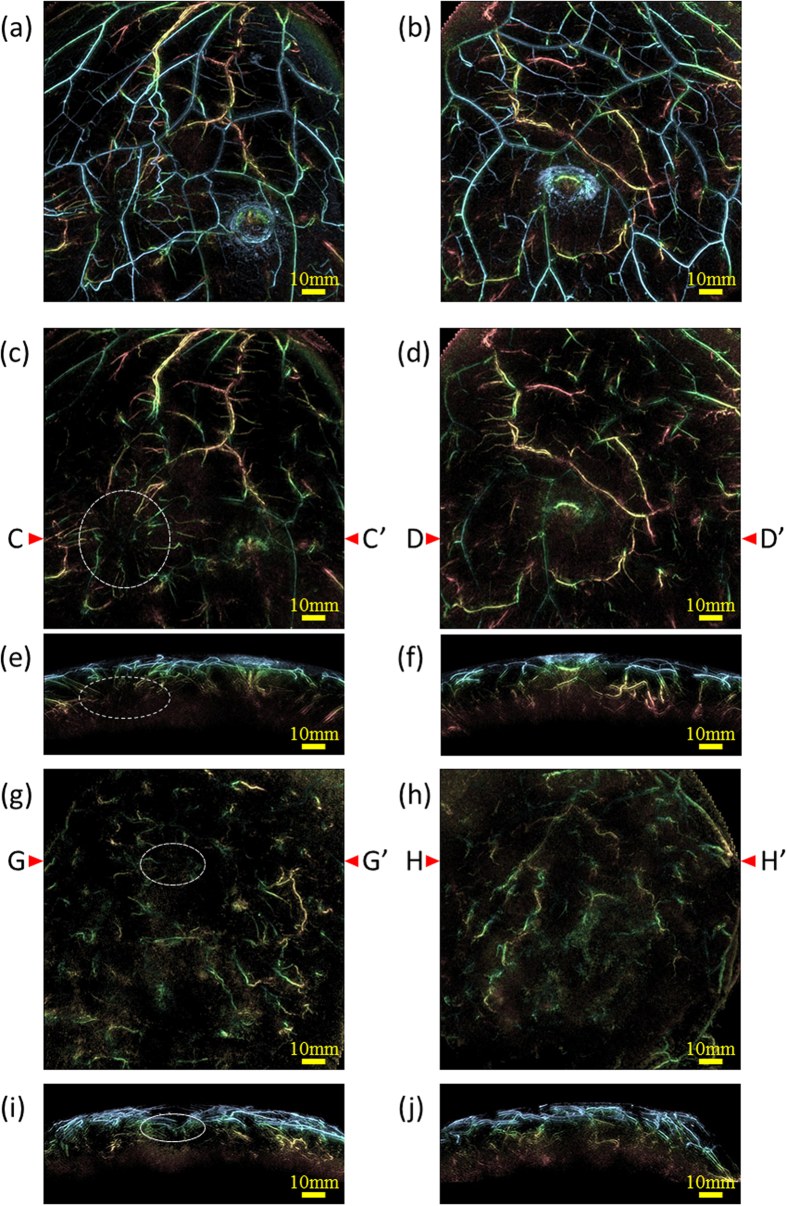
Examples of MIP images of PAI for comparison of amounts of blood vessels between the affected side and the contralateral side. The color of PA signal corresponded to the depth scaled by Fig. 1(g). Case 3 (Fig. 3(a–f)): A 40-year-old woman with IBC in upper-outer area of right breast. The tumor was 47 mm in diameter. (**a**) MIP image of the affected side and (**b**) MIP image of the contralateral side. Significant difference of blood vessel amounts was hardly seen between (**a**) and (**b**) due to many signals in both pictures. MIP image of (**c**) the affected side and (**d**) the contralateral side after eliminating the PA signals from subcutaneous blood vessels by a depth of 4 mm. The position of the tumor was indicated by a white dashed circle. Specific structure of blood vessels could be seen around the tumor in (**c**), whereas not in (**d**). Tumor-related blood vessels were made more visible by eliminating subcutaneous signals in coronal view. (**e**) and 3 (**f**) are slab MIP images observed from the Axial plane of (**c**) and (**d**). The section line was set on C - C’ corresponding to the tumor position and on D - D’ on the contralateral side. The width of each slab was set to 38 mm around the cross section line. Case 4 (Fig. 3(g–j)): A 44-year-old woman with multiple IBCs in upper area in left breast. MIP image of (**g**) the affected side and (**h**) the contralateral side after eliminating the PA signals from subcutaneous blood vessels by a depth of 4 mm. The position of the tumor was indicated by a white dashed-dotted circle. More signals were seen in the affected side than the contralateral side and centripetal blood vessel toward the tumor could be seen in (**g**). (**i**) and (**j**) are slab MIP images of the axial views of (**g**) and (**h**), of which the cross-section line was G-G’ and H-H’, respectively. The width of each slab was set to 38 mm around the cross section line.

First, we compared the morphology and the quantity of the PA signals between affected breast and the contralateral breast. As a result, there were more PA signals considered to be blood vessels in the affected breast than that in the contralateral breast in most cases. Figure 3 shows the examples of the comparison in two IBC cases; one case (Case 3) is (a) – (d), the other (Case 4) is (e) and (f). Figure 3(a) is a MIP image of the whole breast of the affected breast, and (b) is that of the contralateral breast. It was difficult to perceive the difference since there were many subcutaneous blood vessels. Figure 3(c and d) are the images of which signals from subcutaneous blood vessels were eliminated from (a) and (b), respectively. We could find the distinguishing signals in the left lower of Fig. 3(c), which was not seen in (d). Figure 3(e and f) are the MIP images of the other case of which PA signals from subcutaneous blood vessels were eliminated. We can see more PA signals in Fig. 3(e) of the affected breast than that in (f). The total numbers of these trunks and branches of blood vessels in Fig. 3(c,d,g) and (h) by means of manual count conducted by two experts were 185, 151, 121 and 99, respectively. Although there were individual differences in the absolute value of the number of blood vessels, it is sure that the morphological variation on the blood vessel occurs in the affected breast.

**Figure 4:**
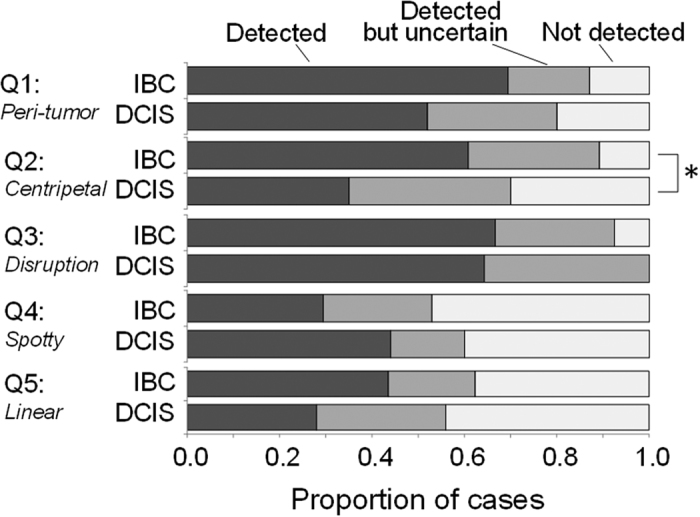
Comparison of clinical findings between IBC and DCIS cases. The following five questions (Q) were assessed. Q1: Are peritumoral PA vasculature signals present? Q2: Is there centripetal vasculature directed toward the tumor? Q3: Is vessel disruption or rapid narrowing present at the boundary of the tumor? Q4: Is a spotty signal present inside the tumor? Q5: Is a vascular-like linear signal present inside the tumor? A statistically significant difference between DCIS and IBC was denoted by an asterisk.

We also compared clinical findings between the DCIS and IBC cases based on the assessment of five questions. The questions and their results were summarized in Fig. 4. Peritumoral vasculature was detected in 86% of all cases (including uncertain cases), suggesting that PAMMG is generally able to image tumor-related blood vessels. In IBC cases, most tumor-related blood vessels were centripetally directed toward the tumor, and 93% of centripetal blood vessels appeared to be disrupted or rapidly narrowed at the tumor boundary.

A significant difference was seen between DCIS and IBC for Question 2; IBC cases tended to have a centripetal blood vessel structure. No significant differences were detected between DCIS and IBC for the other questions; however, there was a trend toward increased identification of intratumoral spotty signals among DCIS cases, and of intratumor vascular-like linear signals among IBC cases. Additional statistical subanalysis revealed no significant difference between luminal and other subtypes of IBC.

**Figure 5:**
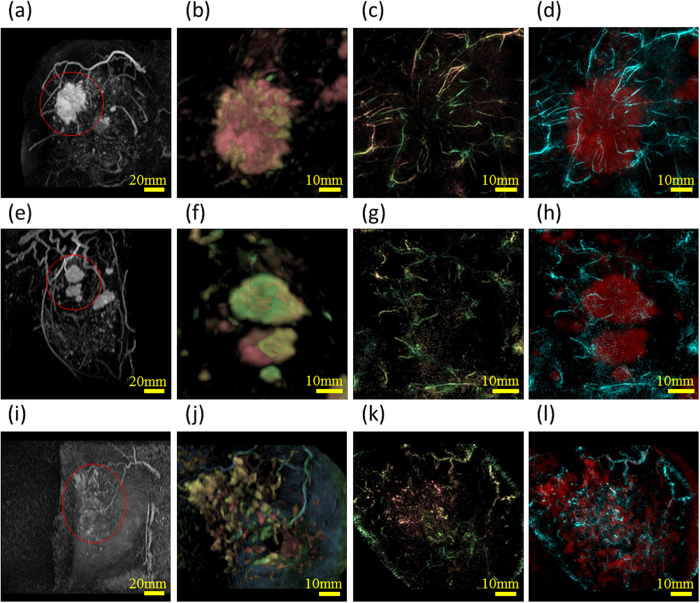
Examples of peritumoral images from three cases. Figures of the first column from the left are the original MR images. Each lesion is indicated by a red circle. Those of the second column are the enlarged MR images around the lesion after deforming into the shape to PA images. The third and the fourth are original PA images, and fusion images of PA (cyan) and MR (red), respectively. These figures are after eliminating the PA signals from subcutaneous blood vessels by a depth of 4 mm. Case 3 (**a**) –(**d**): A 40-year-old woman with IBC. The tumor is 47 mm in diameter. Tumor-related blood vessels seem to converge from the normal breast tissue toward the center of the tumor, becoming drastically narrower at the tumor edge and nearly vanishing near the center. Case 4 (**e**) – (**h**): A 44-year-old woman with multiple IBCs. Tumor-related blood vessels seem to converge toward the center of the tumor in several masses. Case 5 (**i**) – (**l**): A 46-year-old woman with DCIS in upper-outer area in right breast. Non-mass enhancement was seen by MRI almost in the center of the breast. Centripetal blood vessels are hardly evident around the tumor. Spotty signals are seen in the PAI.

Examples of MR, PA and their fusion images are presented in Fig. 5. Images in the first column from the left are the original MR images. Those in the second row are MR images after deforming into the shape of PA images, which are colored according to the image depth. Those in the third row are PA images, and the fourth fusion images of PA and MR. The solid mass in MR images corresponds to tumor. Signals from Hb inside the blood vessels could be seen in the PA images. Centripetal blood vessels were visible toward the middle of the tumor mass in the MR-PA fusion images in Cases 3 and 4. In addition, some blood flow was detected by superb microvascular imaging (SMI^18^; data not shown). The corresponding point count and accuracy of the breast deformation were 162 and 2.6 mm in Case 3 and 240 and 2.4 mm in Case 4, respectively.

In the PA image of DCIS Case 5, spotty signals were observed and centripetal blood vessels were hardly evident around the tumor. The corresponding point count and accuracy of the breast deformation were 64 and 1.6 mm, respectively.

**Figure 6:**
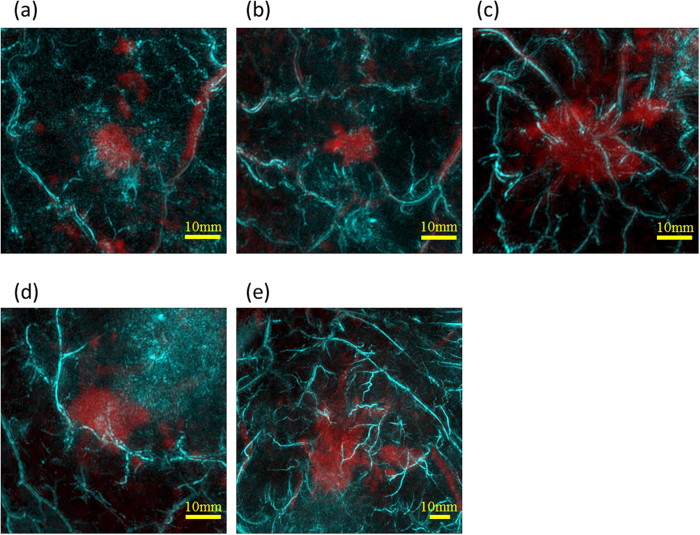
The other examples of peritumoral images from five cases. All figures are fusion images of PA (cyan) and MR (red). Case 6 (**a**): A 65-year-old woman with IBC in upper area of left breast. The tumor was 13 mm in diameter. Case 7 (**b**): A 82-year-old woman with IBC in upper area of left breast. The tumor was 14 mm in diameter. Case 8 (**c**): A 65-year-old woman with IBC in lower-outer area of right breast. The tumor was 28 mm in diameter. Case 9 (**d**): A 45-year-old woman with IBC in lower-inner area of left breast. The tumor was 13 mm in diameter. Case 6 (**e**): A 36-year-old woman with IBC in upper area of left breast. The tumor was 30 mm in diameter. The centripetal blood vessels were observed in all cases.

Regarding other cases of IBC, images after fusion of MR and PA are shown in Fig. 6. Breast deformation accuracy was less than 3 mm. In each case, we could see centripetal blood vessels. In addition, the vessel disruption or rapid narrowing could be seen at the boundary of the tumor in many cases.

**Figure 7:**
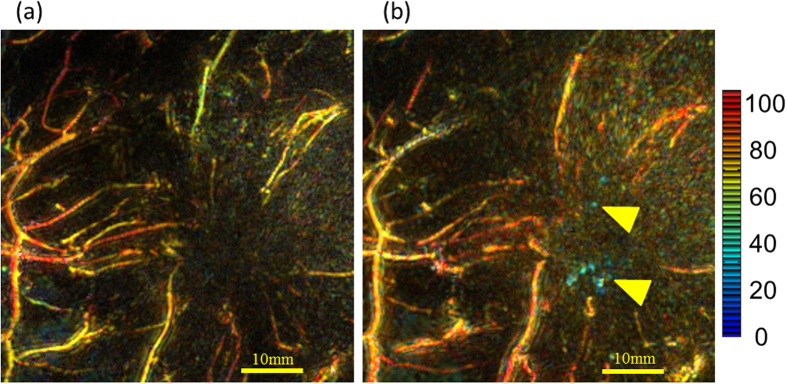
Enlarged images of the peritumoral region in Case 3 from Figure 5. The image intensity represents the measured absorbance coefficient at 795 nm, which corresponds to the amount of Hb present. S-factor values corresponding to the SO_2_ value of Hb are indicated according to the color bar on the right; red indicates close to 100% Hb saturation, whereas blue indicates close to 0%. (**a**) Peritumoral S-factor image obtained by PAM-03 before chemotherapy and (**b**) after chemotherapy. The amount and intensity of intratumoral signal increased after chemotherapy, and the S-factor values were low, indicating hypoxia. Yellow arrows indicate hypoxic spotty signals in the tumor.

It is also possible to observe changes in the PA images of tumor-related blood vessels before and after systemic anticancer treatments such as chemotherapy and anti-Human epidermal growth factor receptor 2 (HER2) therapy. Figure 7 shows changes in a peritumoral PA image before and after taxane-containing chemotherapy in the same patient depicted in Figs 3 and 4, Case 3. Fine intratumoral blood vessels were observed by PAI after chemotherapy, although no significant change in tumor size was detected by US. Slight changes in blood flow were also observed by SMI (data not shown). Taken together, these observations suggest that intratumoral blood flow increased after chemotherapy. Color images depicting S-factor around the lesion before and after chemotherapy (shown in the same figure) indicate that chemotherapy affected not only the amount of blood flow, but also the S-factor value. These results suggest that chemotherapy may affect tumor blood flow and vessel function even without changing tumor size, and that normalization of tumor vasculature may be driven by chemotherapy.

## Discussion

In this study, three-dimensional high-resolution PA images were obtained using the PAM-03 system, allowing the noninvasive visualization of fine vasculature. Most of the blood vessels observed in MRI could be imaged by PAI; furthermore, the blood-vessel structure and morphological characteristics imaged by PAM-03 were more detailed.

PAI has been applied to animal experimental models to visualize fine microvessels however the depth performance was pointed out as a limitation. This report showed the depth performance of PAM-03 with HDA technology based on the continuity from subcutaneous blood vessels. Even though results of 24 mm or 27 mm may be underestimated since there were more unconnected linear signals in deeper area, an imaging capability of 24 mm corresponds to more than 87% of the volume of a 38-mm breast cup; thus, the assessment of most breast lesions is considered feasible using PAM-03. The further improvement of depth performance will be a topic of future study.

We could see the difference of blood vessel quantities and shapes between the affected breast and the contralateral breast other than the subcutaneous blood vessels. It will be interesting to analyze the difference of fine blood vessels between both breasts to diagnose in early stage of breast cancer.

In addition, we developed a technique to deform the breast shape of an MR image to correspond to that of a PA image, which facilitates the clinical application of PAI by enabling the precise visualization of tumor location on PA images. MR image deformation was carried out by overlaying the major corresponding blood vessels between MRI and PAI. On average, the accuracy values of the breast deformation were at most 3 mm.

These technological advances helped clarify several unique features of human tumor-related blood vessels. Even in healthy breast, unconnected blood vessels were often seen by the technical issue such as a limited view problem or lack of sensitivity. However, we could see the specific shape and amount of blood vessels in the affected breast. Notably, a significant difference in peritumoral vascular density was identified in most IBC cases by comparing the affected breasts with the healthy contralateral breasts. Peri- and intratumoral PA signals—in particular, centripetal vasculature directed toward the tumor, and disruption or rapid narrowing of vessels at the tumor boundary—were clearly detected. Vascular-like linear signals and spotty signals inside the tumors were also often seen. Although it has recently become possible to characterize the superficial vasculature using microscopic technologies, it is still difficult to reconstruct three-dimensional vascular structures at deeper tissue levels. It would therefore be remarkable to obtain stereoscopic spatial images of the tumor-related vasculature in primary breast cancer.

The presence of centripetal vasculature signal directed toward the tumor was detected in 61% of IBC cases, compared with only 35% of DCIS cases. Although further analysis is needed, these characteristics may help distinguish between DCIS and IBC lesions. We examined criteria consisting of five questions to assess tumor-related blood vessels in this study; however, new approaches, such as computer-associated learning programs or intelligence systems, will also be helpful to realize more precise diagnosis with PAI in the near future.

The acquisition of PA data using two wavelengths allowed us to assess Hb saturation (SO_2_). The accuracy of the calculated SO_2_ has been questioned because of factors such as uncorrectable motion artifacts and lack of system accuracy; we therefore considered it as a relative value, the S-factor, which is also expected to provide meaningful information.

Interestingly, this study demonstrated that tumor-associated vessels—particularly those present inside the tumor—became visible after preoperative systemic chemotherapy, despite the fact that no change in tumor size was detected by ultrasound examination. Although this effect may have resulted from morphological changes in the vasculature, it is also possible that pre-existing tumor blood vessels became visible because of increased blood flow due to chemotherapy-mediated lowering of interstitial pressure. This could promote the normalization of tumor vasculature, which has been postulated as a mechanism of action of anticancer treatments—including not only chemotherapy, but also molecular targeting therapies such as anti-HER2 antibody treatment. In fact, our observations suggested that S-factor was affected by chemotherapy. The ability to visualize the S-factor, a metric of the oxygenated or reductive status of Hb associated with hypoxic conditions in the tissue microenvironment, is feature of optical imaging technology and its high resolution imaging is the greatest merit of PAI with HDA. The observation of intratumoral blood flow and Hb oxygenation status would give a more precise understanding of the tumor microenvironment, and noninvasively aid in monitoring anticancer treatment-elicited changes in a clinical setting.

In this study, we demonstrated that the effectiveness of PAMMG imaging in deeper tissue zones, such as those over 30 mm in depth from the skin surface, is limited. Because completion of a PAMMG scan with two wavelengths using our current technique takes about 4 minutes, the body motion of patients often causes degradation of image quality and S-factor accuracy even in the use of the body motion compensation. Even though the body motion correction processing was employed, the post processing has a limited effect. Further technological advances should be incorporated to speed up data acquiring and processing and improve three-dimensional resolution at deep tissue levels.

In summary, high-resolution vascular images associated with malignant breast cancer were obtained using a new PAMMG system with a hemispherical detector array. This system allowed the visualization of fine vasculature that is not visible on standard contrast-enhanced MRI. The MR-PA fusion images enabled us to overlay tumor-related vasculature onto tumor mass images. With these technologies, we could analyze the details of tumor-related vascular structures in human breast cancer. The oxygen saturation status of Hb was also visualized using two different wavelengths, helping to more precisely characterize the tumor microenvironment in a noninvasive manner. This new function images may lead to promote early detection of noninvasive cancers in the harmonization with conventional modalities.

## Materials, Patients and Methods

### Device configuration

We used the PAM-03 system with HDA, which was made by Canon Inc. (Japan) in collaboration with Optosonics Inc. (USA). A photograph and schematic illustration of PAM-03 are given in Figure S1(a). Detailed configurations have been already reported^8^, thus rough configurations and the modification points are described as follows.

The system was modified by reducing the depth of the breast-holding cup from 50 mm to 38 mm in consideration of the average breast size of Asian women (Figure S1(b)). In addition, we utilized a laser capable of irradiating at two different wavelengths of 755 and 795 nm in order to evaluate Hb saturation index of the blood vessels, which we call S-factor.

The detector array was scanned continuously in a spiral pattern within a plane. The spiral scanning pattern was modified to gradually heighten the density of data acquisition points as the scan approached the center of the spiral scan as described in Figure S1(c), in order to improve the image quality at the center of the breast cup which corresponded to the deep tissue.

A Q-switched alexandrite laser with selectable wavelengths of 755 and 795 nm was used. The laser energy used in PAM-03 was approximately 200 mJ/pulse, which were constant for both wavelengths. A diameter of light illumination was 60 mm diameter at the surface of the breast cup. The maximum light energy was set to less than 10 mJ/cm^2^, which is half the maximum permissible exposure recommended by the American National Standards Institute.

A piezoelectric zirconate titanate transducer was used to detect the PA signal. The HDA contained 512 elements on its inside surface, each a circle 3 mm in diameter. The central frequency was 2 MHz.

The imaging area for each laser shot was just in range of the cylinder’s 30-mm radius. To enhance the imaging area, the HDA was spirally scanned in the horizontal plane; this allowed the imaging area to be selectable at radii of 50, 70, and 100 mm. The data acquisition number was also selectable at 1024 and 2048 for one scan, corresponding to 51.2 s and 102.4 s of data acquisition time, respectively. Because measurement at two wavelengths required double the measuring time, the actual total operating time was about 4 minutes for a data acquisition number of 2048.

According to our imaging result of full width at half maximum using a spherical phantom with a diameter of 0.3 mm, those along x and y directions were 0.57 mm, and that along z direction was 0.37 mm, respectively.

### Patients

Patients with primary breast lesions who met the inclusion criteria and provided written consent were enrolled in the study. Inclusion criteria were as follows: 20 years old or older at the time of diagnosis, and Eastern Cooperative Oncology Group Performance Status of 0 or 1. The exclusion criteria included pregnancy, suspicion of pregnancy, ongoing photodynamic therapy with photosensitizing agents such as Photofrin, breast implants, cardiac pacemaker, and any other conditions that made the patient unsuitable for participation in the study according to the clinical investigators. A total of 30 patients were considered for the study between December 2014 and December 2015 at Kyoto University Hospital, Japan. All patients had undergone routine radiological (XMMG, US, and/or MRI) and histological diagnosis (core biopsy) prior to surgery. The present study was approved by the Ethics Committee of the Kyoto University Graduate School of Medicine (C 789) and written informed consent was obtained from all patients. This study was conducted in accordance with the Declaration of Helsinki.

### PAMMG data acquisition

Both of each patient’s breasts were inserted in body-temperature water and scanned at wavelengths of 795 nm and then 755 nm. The affected breast was scanned first, followed by the contralateral side. Lesions were identified primarily by US.

PA image reconstruction was carried out using a universal back-projection algorithm^19^. At this time, the PA image was reconstructed in consideration of both the average sound speed of the patients’ breast and that of water for impedance matching, thereby reducing the degradation of the image resolution in the living body. The light absorption coefficient (μ_a_) and Hb saturation index (S-factor) were calculated using a commonly used method^12^,^13^. The variations of light fluence in tissue among each patient were compensated according to the calculated result of diffusion equation^13^. For the S-factor images in this paper, the S-factor value and the absorption coefficient at 795 nm were indicated by hue and intensity, respectively.

In calculating the μ_a_ and S-factor images, two kinds of three-dimensional body motion correction methods were employed; one is a body motion correction between different laser pulses (p-BMC) for μ_a_ image, the other is that between two different laser wavelengths of 755 nm and 795 nm (w-BMC) for S-factor image. p-BMC is a correction method of the body motion during a single spiral scan of HDA with 2048 (or 1024) laser shots. Each PA image was reconstructed using corresponding laser shot, which we call “pulse image”. We estimated the movement vectors of body motion between plural pulse images by using the sum of squared distance as a similarity measure. The final image after the body motion correction in a single wavelength was obtained by integrating all pulse images corrected by estimated movement vectors of body motion. w-BMC is a correction method of the body motion between two wavelengths’ images. We conducted w-BMC by using the free-form deformation (FFD) method^20^ to deform the breast shape of 755 nm image toward that of 795 nm image. The spacing of the control points of FFD was 15 mm for all axes.

After obtaining the μ_a_ image, the data in the vicinity of the subcutaneous tissue was deleted by image processing as necessary. In order to obtain information on the body surface, a cloth simulation method was applied^21^. By covering the volume data of the PA image with a virtual soft cloth with no gaps, the position information of the virtual cloth was regarded as information of the body surface. The volume data was trimmed from the body surface position to an arbitrary depth to simplify the image interpretation of deeper tissues.

### MRI data acquisition

Breast MRI was obtained with a 3.0 T scanner (MAGNETOM Trio, A Tim System, Siemens AG, Germany) with a dedicated 16-channel breast array coil.

Fat-suppressed T1-weighted dynamic contrast-enhanced (DCE) images were obtained pre-contrast and then at 1–2 min (early) and 5–6 min (delayed) after gadolinium injection. Whole-breast axial scanning at a high temporal resolution was performed for 1 min (3D-VIBE: TR/TE 3.70/1.36 ms, FA 15 and FOV 330 mm × 330 mm, matrix 384 × 346, thickness 1.0 mm). Subtraction images were computed pixelwise by subtracting the signal intensity of pre-contrast images from that of early post-contrast images. Gadoteridol (ProHance, Eisai Inc., Tokyo, Japan) was power injected at a dose of 0.2 mL/kg and a speed of 2.0 mL/s, then flushed with 20 mL saline at the same rate.

In five patients, breast MRI was obtained using a different 3.0 T scanner with slightly different parameters. The quality of pre- and post-contrast fat-suppressed T1-weighted DCE images was checked by radiologists to ensure that quality was equivalent to that of images obtained with the scanner described above.

### Image fusion with deformed MRI

There are reports that analyze tumors by simply superimposing PA and MR^22^. However, simple superposition is considered unsuitable for breast cancer diagnosis with different breast shape depending on the type of modality. We adapted the breast image deformation method to compare the image acquired from PAMMG with that from MRI and make a fusion image of both modalities. Blood vessels can be observed in pre-contrast and early post-contrast MR subtraction images, as well as PA images. For the first time in the field of breast cancer diagnosis, we manually identified common features such as blood-vessel branching points in both modalities; these were called “corresponding points,” then we deformed the whole breast shape in three dimensions to match each corresponding point by applying the thin plate spline (TPS) method^23^. We analyzed PA images as well as fusions of deformed breast MR images and PA images.

The precision of breast deformations was evaluated using the leave-one-out method, as follows. One pair of corresponding points was selected to evaluate precision; the breast deformation was then carried out again, this time omitting the selected points. The target registration error was calculated from the difference between deformations with and without the selected points^24^. This calculation was performed for all identified corresponding points in the breast, and the median value was adopted as a precision value for the image fusion.

### Mammary ultrasound diagnosis

Before PAMMG scanning, a mammary ultrasound examination was carried out using an Aplio 500 ultrasound machine (Toshiba, Japan). Standard B-mode images and high-resolution Doppler images (superb microvascular imaging, SMI) were obtained^18^.

### Pathologic diagnosis

After surgery, excised specimens were sectioned at 5-mm intervals perpendicular to the longest axis of the specimen, and permanent pathologic analysis was performed on formalin-fixed paraffin-embedded tissues by conventional hematoxylin-eosin staining. For invasive breast carcinoma (IBC) and ductal carcinoma *in situ* (DCIS), estrogen receptor and progesterone receptor status were confirmed by immunohistochemistry (IHC). HER2 status was confirmed by IHC or fluorescent *in situ* hybridization. Ki-67 immunostaining was performed using the MIB1 monoclonal antibody (Dako, Copenhagen, Denmark) for IBCs. Histological grade for IBCs was calculated by an experienced breast pathologist according to the General Rules for Clinical and Pathological Recording of Breast Cancer, 15th edition, based on scores for nuclear pleomorphism, tubule formation, and mitotic count.

### Assessment of PAMMG images

PA images were assessed by five breast diagnosis specialists: two radiologists, and three breast surgeons specializing in breast disease. Blood vessel PA images were assessed according to multiple questions: 1) Are peritumoral PA vasculature signals present? 2) Is there centripetal vasculature directed toward the tumor? 3) Is vessel disruption or rapid narrowing present at the boundary of the tumor? 4) Is a spotty signal present inside the tumor? and 5) Is a vascular-like linear signal present inside the tumor? The tumor boundary was defined by the MR-PA image fusion. The five readers answered each question with one of three ratings: “not detected,” “detected but uncertain,” or “detected.” Question 2 was assessed if the answer to Question 1 was “detected” or “detected but uncertain,” and Question 3 was assessed if the answer to Question 2 was “detected” or “detected but uncertain.” Scores were compared between IBC and DCIS using the Wilcoxon rank-sum test. P values less than 0.05 were considered statistically significant. Scores were also compared between the luminal subtype and other types of IBC.

## Additional Information

**How to cite this article:** Toi, M. *et al*. Visualization of tumor-related blood vessels in human breast by photoacoustic imaging system with a hemispherical detector array. *Sci. Rep.* **7**, 41970; doi: 10.1038/srep41970 (2017).

**Publisher's note:** Springer Nature remains neutral with regard to jurisdictional claims in published maps and institutional affiliations.

## Supplementary Material

Supplementary Video 1

Supplementary Video 2

Supplementary Video 3

Supplementary Video 4

Supplementary Video 5

Supplementary Document
