# Supplementary material for: Visualization of tumor-related blood vessels in human breast by photoacoustic imaging system with a hemispherical detector array
Source: Sci Rep. 2017 Feb 7;7:41970. doi: 10.1038/srep41970 (PMC5294462; doi:10.1038/srep41970)

Visualization of tumor-related blood vessels in human breast by photoacoustic imaging system with a hemispherical detector array

Authors:

M. Toi[1*], Y. Asao[1,5], Y. Matsumoto[1], H. Sekiguchi[2], A. Yoshikawa[1], M. Takada[1], M. Kataoka[2], T. Endo[5], N. Kawaguchi-Sakita[1], M. Kawashima[1], E. Fakhrejahani[1], S. Kanao[2], I. Yamaga[1], Y. Nakayama[1], M. Tokiwa[1], M. Torii[1], T. Yagi[5], T. Sakurai[3], K. Togashi[2] and T. Shiina[4]

Affiliations:

[1]Department of Breast Surgery, Graduate School of Medicine, Kyoto University, 54 Shogoin-Kawaharacho Sakyo-ku, Kyoto 606-8507, Japan

[2]Department of Diagnostic Imaging and Nuclear Medicine, Graduate School of Medicine, Kyoto University, 54 Shogoin-Kawaharacho Sakyo-ku, Kyoto 6068507, Japan

[3]Department of Diagnostic Pathology, Graduate School of Medicine, Kyoto University, 54 Shogoin-Kawaharacho Sakyo-ku, Kyoto 606-8507, Japan

[4]Department of Human Health Science, Graduate School of Medicine, Kyoto University, 53 Shogoin-Kawaharacho Sakyo-ku, Kyoto 606-8507, Japan

[5]Medical Imaging System Development Center, Canon Inc., 3-30-2 Shimomaruko, Ohta-ku, Tokyo 146-8501, Japan

*To whom correspondence:*toi@kuhp.kyoto-u.ac.jp

Supplementary figures:

Fig. S1. (a) Photograph of PAM-03. The patient lies prone on the bed and placing one breast in a spherically shaped cup indicated by yellow arrow. (b) A schematic illustration of the breast holding cup and the hemispherical detector array (HDA). (c) The spiral scan pattern used in this report, which was modified to gradually heighten the density of data acquisition points as the scan approached the center of the spiral scan.

Fig. S2. An example of image improvement by body motion correction during a single spiral scan (p-BMC). (a) Without p-BMC processing and (b) with p-BMC processing.

Supplementary movies:

Fig2a_movie_1.avi. The movie of rotating the MIP image of Case 1 as shown in Figure 2 (a).

Fig2b_movie_1.avi. The movie of rotating the MIP image of Case 2 as shown in Figure 2 (b).

Fig5d_movie_1.avi. The movie of rotating the fusion image of PA (cyan) and MR (red) of Case 3 as shown in Figure 5 (d).

Fig5h_movie_1.avi. The movie of rotating the fusion image of PA (cyan) and MR (red) of Case 4 as shown in Figure 5 (h).

Fig5l_movie_1.avi. The movie of rotating the fusion image of PA (cyan) and MR (red) of Case 5 as shown in Figure 5 (l).

Figure S1

(a)

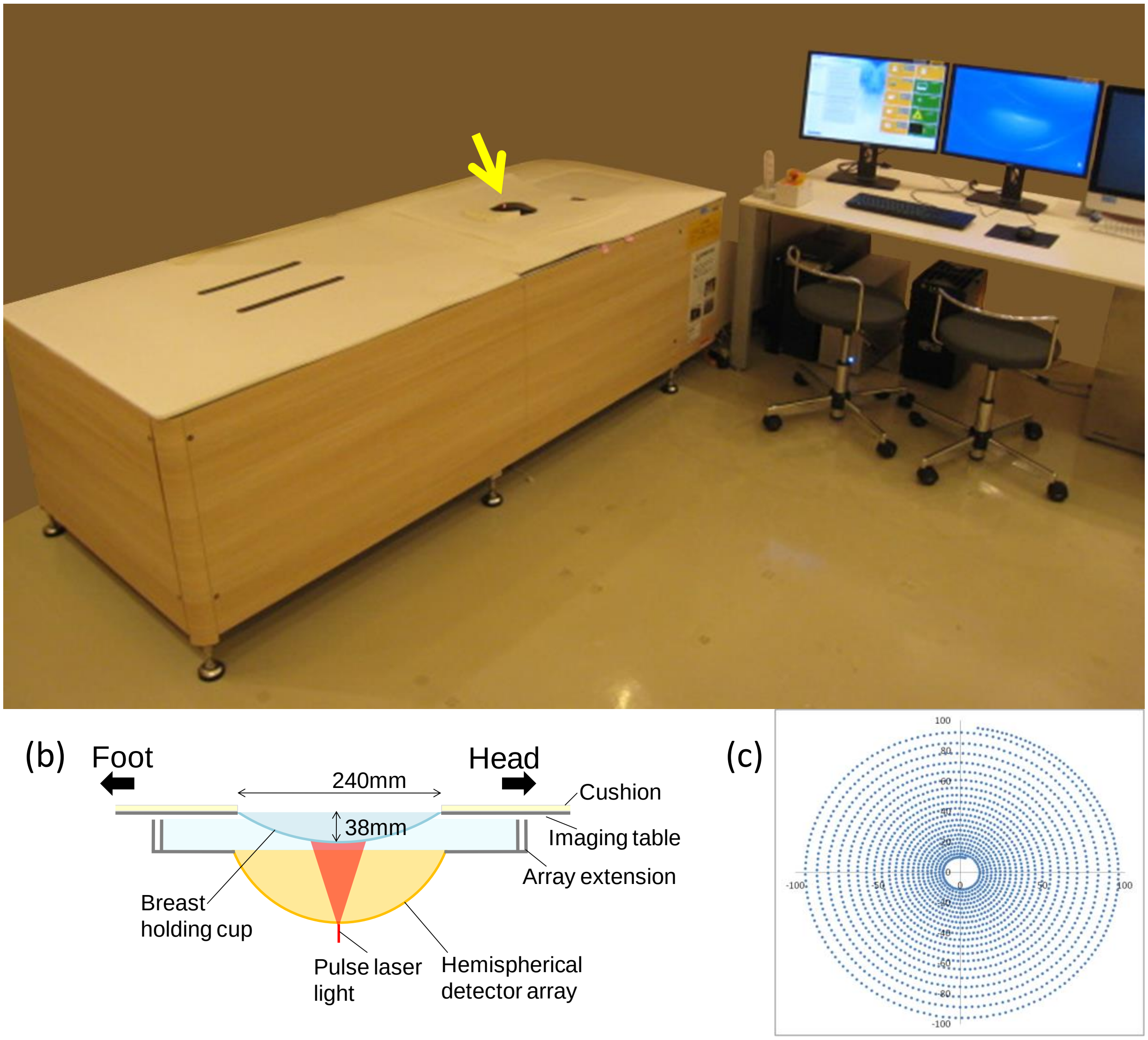
(b)
Foot
Head
240mm
38mm
Cushion
Imaging table
Array extension
Breast holding cup
Pulse laser light
Hemispherical detector array
(c)
100
-100
0
50
-50
100
-100

Figure S2

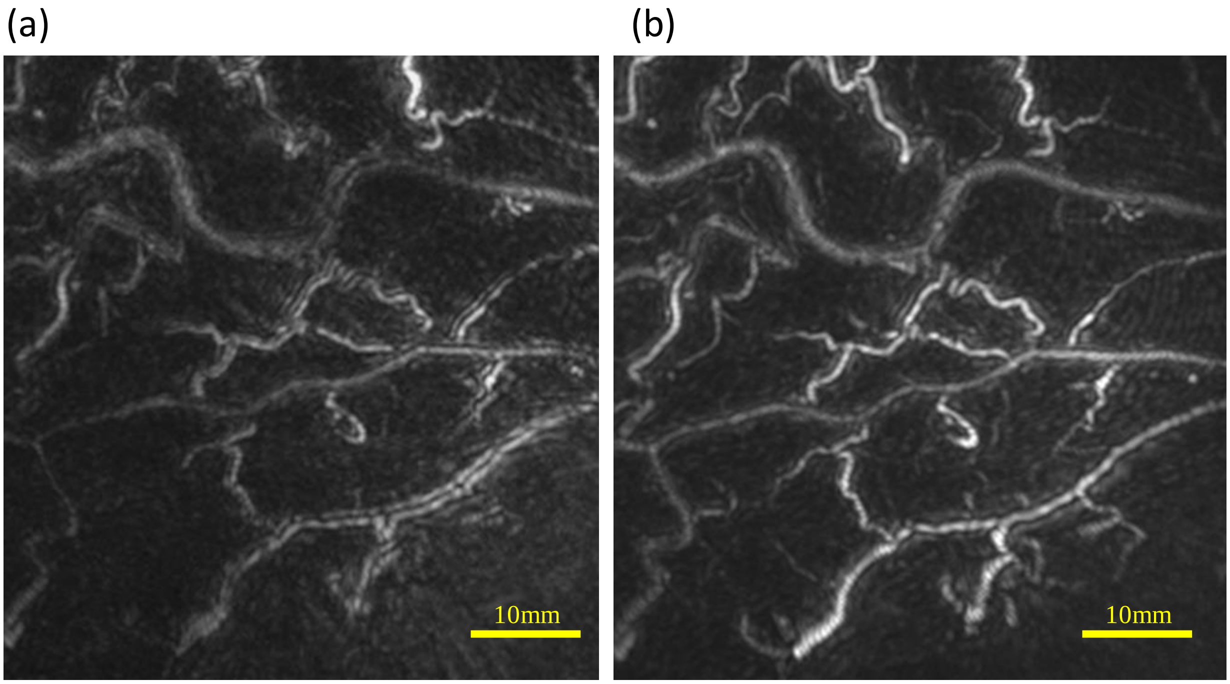

Supplement: Supplementary Document [file srep41970-s6.pdf]
